# 柚子皮生物炭质用于河水中苯系物的固相微萃取

**DOI:** 10.3724/SP.J.1123.2022.02006

**Published:** 2022-11-08

**Authors:** Jingjing CHEN, Zhuoran ZHANG, Jianfeng YU, Shiming TANG, Bingwen CUI, Jingbin ZENG

**Affiliations:** 中国石油大学(华东)化学化工学院, 山东 青岛 266580; College of Chemistry and Chemical Engineering, China University of Petroleum, Qingdao 266580, China

**Keywords:** 气相色谱-火焰离子化检测, 固相微萃取, 热解温度, 柚子皮生物炭质, 苯系物, 河水, gas chromatography-flame ionization detection (GC-FID), solid phase microextraction (SPME), pyrolysis temperature, pomelo peel biochar (PPB), benzenes (BTEX), river water

## Abstract

苯、甲苯、乙苯和二甲苯(邻二甲苯、间二甲苯、对二甲苯)组成的苯系物(BTEX)是炼油厂和石化厂等工业园区普遍制造和排放的碳氢化合物,具有一定的毒性和致癌作用,对生态环境和人类健康造成极大威胁。研究以低成本、绿色且富含木质素和含氧官能团的柚子皮作为植物原料,在有限氧条件下采用程序升温热解法制备了柚子皮生物炭质吸附剂,通过N_2_吸附-脱附等温线和孔径分布图对不同热解温度下制备的柚子皮生物炭质吸附剂的孔隙结构进行了考察。结果表明:在1000 ℃热解温度下制得的柚子皮生物炭质具有更高的比表面积(749.9 m^2^/g)、更大的孔体积(0.42 cm^3^/g)、更集中的孔径分布(2~3 nm)。将吸附剂通过溶胶-凝胶法(sol-gel)涂覆在铁丝上制成固相微萃取纤维,与气相色谱-火焰离子化检测器(GC-FID)相结合,对影响萃取和分离BTEX的条件进行优化,建立了用于BTEX检测的高灵敏度分析方法。方法具有检出限低(0.004~0.032 μg/L)、线性范围宽(1~100 μg/L)、线性关系好、萃取效率高(约为商品化涂层聚二甲基硅氧烷(7 μm)的2.9~18.3倍)等优势。此外,应用该方法已成功在河水样本中检测出了乙基苯(4.80 μg/L),邻二甲苯(3.00 μg/L)和对二甲苯、间二甲苯(2.46 μg/L)。最后将该方法应用于河水样本的加标试验中,得到了满意的回收率(75.7%~117.6%)。实验结果表明所建立的分析方法可实现对环境水样(河水)中BTEX的低成本、高灵敏度检测。

## Abstract (en)

Pomelo peel, as a by-product of pomelo consumption, is rich in various fiber and functional compounds. The utilization of the valuable components found in pomelo peel may mitigate environmental concerns. In this study, pomelo peel rich in lignin and oxygen-containing functional groups was used to prepare pomelo peel biochar (PPB) via temperature-programmed pyrolysis at different temperatures (800 ℃ and 1000 ℃). Their structures were investigated by N_2_ adsorption-desorption isotherms and BJH pore size distribution. The results showed that PPB1000 (pomelo peel biochar prepared at 1000 ℃) had a higher specific surface area (749.9 m^2^/g), larger pore volume (0.42 cm^3^/g), more concentrated pore size distribution (2-3 nm), and better adsorption performance than commercial activated carbon. PPB1000 exhibited excellent capability to capture benzenes (BTEX, including benzene (B), toluene (T), ethylbenzene (E), and xylene (X)) through hydrogen bonds, *π-π*, and electrostatic interactions. Additionally, their honeycomb porous structure could provide additional adsorption sites and material transport paths. PPB1000 was coated on iron wire using the sol-gel method to prepare chemically and mechanically stable solid phase microextraction (SPME) fibers. By combining PPB1000-based SPME analysis with gas chromatography-flame ionization detection (GC-FID), an effective method was developed for the extraction and determination of BTEX. The optimized method had low LODs (0.004-0.032 μg/L), wide linear range (1-100 μg/L), and good linear relationship (determination coefficients, *r*^2^≥0.9919). The RSDs of the intra-batch (*n*=6) and inter-batch (*n*=5) precisions were 1.04%-6.56% and 1.03%-12.42%, respectively. The method validation results showed that PPB1000 had good stability. Compared with the commercial reagent polydimethylsiloxane (7 μm), PPB1000 had a higher extraction efficiency. When applied to the analysis of BTEX in natural water samples, trace levels of ethylbenzene (4.80 μg/L), *o*-xylene (3. 00 μg/L), and *m*-xylene and *p*-xylene (2.46 μg/L) were detected. Recovery tests were performed to validate the reliability of the method, and recoveries were between 75.7% and 117.6%. This effective pretreatment process combined with GC-FID could realize the rapid detection of BTEX and is promising for the analysis of BTEX in complex matrixes in the future.

苯(benzene, B)、甲苯(toluene, T)、乙苯(ethylbenzene, E)和二甲苯(xylene,包括邻二甲苯(*o*-xylene, *o*-X)、间二甲苯(*m*-xylene, *m*-X)和对二甲苯(*p*-xylene, *p*-X))组成的苯系物(benzenes, BTEX)是一类在化学化工厂、炼油厂、发电厂等工业园区普遍使用且具有毒性的碳氢化合物,而意外泄漏、处理不完全和非法排放等事故不可避免地将其引入地表和地下水体中造成水生生态系统受损^[1,2]^。人体长期接触BTEX将会增加器官受损,以及罹患神经认知障碍等身体疾病的风险。因此,BTEX已被纳入我国生活饮用水标准以及水体优先控制污染物监测范围之内^[3]^。为了人体健康和生态安全,迫切需要建立一种简单、低成本、灵敏的分析方法用于实际水体中BTEX的检测。

目前,用于分离、定量检测水体中BTEX的方法主要有气相色谱法(GC)^[4⇓⇓⇓-8]^和高效液相色谱法(HPLC)^[9,10]^。由于BTEX在水体中痕量存在,仪器很难直接对饮用水或地表水中的BTEX进行分离检测。而采用恰当的样品预处理方法可以降低复杂基质的潜在干扰,同时可以富集痕量目标分析物以达到检测需求。近年来,用于水体中提取、富集BTEX的手段主要包括液-液萃取(LLE)、固相微萃取(SPME)和分散固相萃取(DSPE)等^[11,12]^。其中,LLE法存在使用大量有机溶剂、处理过程繁琐等问题;DSPE法存在分析物易损失的问题。而SPME技术与其他分析仪器联用可以做到富集、检测一体化。其中,SPME-GC联用技术,所需样品溶液体积小,分离选择性高,检出限可达到μg/L,是痕量分析领域经典的联用技术之一。

纤维涂层是SPME中最重要的组成部分,是决定方法灵敏性、重现性、使用寿命和实际应用能力的关键。近年来,具有高比表面积、大孔隙率和丰富相互作用位点的固相吸附剂已经广泛用于SPME领域,虽然这些材料表现出良好的萃取效率,但复杂的合成反应或化学功能化是不可避免的。植物源生物质绿色环保且种类繁多,由其制备的生物炭质吸附剂具有良好的物化性质^[13,14]^。目前,制备手段包括热解、水热碳化和微波辅助碳化。其中,热解是在一种廉价、可靠且简单的热化学技术下,即在有限氧供应条件下分解有机物从而获得富碳产品的方法,是研究人员普遍选择的一种制备方式。生物炭质的特性主要受原料性质和热解温度的影响。不同植物源生物炭中木质素、纤维素和无机物的含量、表面官能团的特性以及不同的孔隙结构,决定了它们对于目标分析物具有不同的吸附性能。此外,热解温度会影响孔的形成以及表面物理化学性质(极性、疏水性),对于植物源生物质,完全炭化热解温度需要高于500 ℃^[15]^。

柚子是东亚和东南亚日常消费或果汁/糖等商品生产所需的常见柑橘类水果之一。柚子皮约占新鲜水果重量的30%,处理不当的柚子皮腐烂后还会造成环境污染等问题^[16]^。研究发现:柚皮具有的泡沫状纤维层结构主要由可溶性单糖、不溶性多糖和天然芳香聚合物木质素组成,其中富含羟基、羧基、酚羟基、氨基、磺酸基等众多有机官能团,这些官能团可通过氢键、*π-π*相互作用、静电相互作用吸附有机污染物,同时其具有的蜂窝状多孔结构可增加额外的吸附位点及物质传输路径。因此,柚子皮是一种极具应用前景的生物炭原材料^[17,18]^。

基于此,本研究以柚子皮作为原材料,利用有限氧程序升温热解法制备了热解温度为800 ℃和1000 ℃的柚子皮生物炭质(pomelo peel biochar, PPB)吸附剂。利用扫描电子显微镜、氮气吸附-脱附实验等手段对生物炭质进行形貌和结构表征,表征结果证明在1000 ℃下制备的柚子皮生物炭质内部包含许多由块状物质堆积而成的孔道结构且拥有较大的比表面积。利用溶胶-凝胶(sol-gel)法将其修饰于不锈钢丝表面制成SPME纤维并与GC-FID相结合,经过优化萃取和分离条件,建立了一种适用于检测水体中痕量BTEX的分析方法。

## 1 实验部分

### 1.1 仪器与试剂

GC-2010 Plus气相色谱仪,配有FID检测器、DB-5型毛细管柱(日本Shimadzu公司);加热磁力搅拌器(德国IKA公司);高速万能粉碎机(上海顶帅电器有限公司);微量进样器(上海高鸽工贸有限公司); S-4800型冷场扫描电镜(日本Hitachi公司); Tristar 3000型N_2_吸附仪(美国Micromeritics仪器); NEXUS-670傅里叶变换红外光谱仪(美国Nicolet公司)。

丙酮(纯度99.5%)购自天津化学试剂有限公司;间二甲苯(纯度95%)、邻二甲苯(纯度98%)、对二甲苯(纯度98.5%)、苯酚(纯度99.5%)、乙基苯(ethylbenzene,纯度98.5%)、无水乙醇(纯度≥99.7%)购自国药集团化学试剂有限公司;苯(纯度99.5%)购自天津化学试剂有限公司;甲苯(纯度99.5%)购自利安隆博华医药有限公司;萘(纯度≥99.7%)购自北京化工厂;甲醇(纯度≥99.8%)购自美国飞世尔科技公司。

### 1.2 分析条件

日本岛津DB-5型毛细管柱(30.0 m×0.25 mm×0.25 μm);进样口温度200 ℃,分流进样,分流阀开启时间为3.0 min。色谱柱升温程序:40 ℃保持1 min,以8 ℃/min升温至90 ℃,保持1 min,以30 ℃/min升温至210 ℃,保持1 min。高纯氮气为载气,柱流量为1.2 mL/min,恒压模式;尾吹气流量为30 mL/min。FID温度为250 ℃,氢气流量为40 mL/min,空气流量为410 mL/min。

### 1.3 标准溶液的配制

萘及苯酚标准储备液的配制:量取适量萘及苯酚,分别用水和无水乙醇溶解配制成质量浓度均为100 μg/mL的标准储备液。

BTEX标准储备液的配制:分别准确量取适量苯、甲苯、乙苯、对二甲苯、间二甲苯和邻二甲苯,以甲醇作为溶剂配制成质量浓度为1.0 mg/L的BTEX标准储备液。

将上述配制好的标准储备液避光保存在5 ℃的冰箱中,后续按照实验要求稀释成所需浓度的系列BTEX标准工作溶液。

### 1.4 柚子皮生物炭质的制备

#### 1.4.1 柚子皮粉末的制备

将从中国石油大学(华东)附近市场购买的柚子(福建产)剔除果肉和白色絮状内瓤后,用去离子水彻底清洗柚皮,并切成1×1 cm^2^小块,在60 ℃烘箱中烘干。干燥的柚皮经高速万能粉碎机粉碎、研磨后过60目筛,得到的柚子皮粉末储存于聚乙烯塑料样品袋中备用。

#### 1.4.2 柚子皮生物炭质材料的制备

取适量干燥的柚子皮粉末,置于瓷方舟,然后放置于炭化炉内,N_2_流速设定为50 mL/min,目的是排出炉腔中存在的氧气。同时以5 ℃/min的速率升温至目标温度1000 ℃,热解4 h。所得产物于1.0 mol/L盐酸中处理12 h以去除表面灰分(无机物),然后使用超纯水反复冲洗、抽滤至滤液pH值处于中性范围内,于80 ℃恒温烘箱中烘干,得到黑色粉末状柚子皮生物炭质。

### 1.5 固相微萃取纤维的制备

#### 1.5.1 不锈钢丝载体的预处理

涂覆前,将不锈钢丝一端在超声清洗机中分别用丙酮、甲醇、超纯水超声处理8 min,以去除表面杂质。待自然晾干使用。

#### 1.5.2 溶胶-凝胶溶液的配制

在10 mL离心管中依次加入二甲基二甲氧基硅烷(720 μL)、原硅酸四甲酯(440 μL)和1 mol/L盐酸溶液(40 μL),超声5 min后,以8000 r/min的转速离心5 min,得到溶胶-凝胶溶液。

#### 1.5.3 纤维的制备

准确称取0.1 g柚子皮生物炭质粉末作为涂层材料,同时量取400 μL溶胶-凝胶溶液作为黏合剂于离心管中。将不锈钢丝处理好的一端竖直旋入溶胶-凝胶溶液中并保持1 min左右,取出后立即插入生物炭质粉末中,轻缓旋转使粉末均匀涂覆在不锈钢丝表面,慢慢抖落表面多余粉末;最后将载体置于150 ℃烘箱中干燥35 min,重复上述步骤直至得到10 μm的生物炭质涂层,随后将涂覆有柚子皮生物炭质涂层的不锈钢铁丝安装到微量进样器(5 μL)中并转移至GC进样口,在250 ℃和分流进样条件下老化30 min。

### 1.6 样品预处理

河水样品取自青岛市黄岛区丁家河,首先通过抽滤去除实际样品中存在的泥沙以及植物纤维等杂质,随后利用孔径小于0.22 μm的滤膜对其纯化,得到样品溶液。

### 1.7 固相微萃取过程

准确量取15 mL样品溶液,置于含有0.75 g NaCl的20 mL玻璃小瓶中,将其放置于可控温磁力搅拌器中。萃取时,将涂层完全浸入液面以下,室温下搅拌(1250 r/min)萃取20 min后,将萃取纤维从玻璃瓶中抽出并转移至GC进样口,解吸2 min。

## 2 结果与讨论

### 2.1 热解温度优化及材料表征

热解温度对生物炭质的性质及其吸附性能有很大影响,柚子皮中包含的木质素在500 ℃左右才会被热解,且温度越高,热解越完全,失重越大。此外,已有大量研究表明在较高热解温度下制备的生物炭质材料拥有更大的孔隙度及比表面积,这有利于目标物扩散,加速吸附过程。因而,本实验选取在800和1000 ℃两个热解温度下制备柚子皮生物炭质,考察温度对生物炭质吸附性能的影响,制得的柚子皮炭质粉末分别标记为PPB800、PPB1000。通过N_2_吸附-脱附实验对柚子皮生物炭质材料的比表面积和孔隙率进行了表征,如图1a所示,根据国际纯粹与应用化学联合会分类标准,该曲线属于典型的Ⅳ型等温线,即高温热解下得到的PPB800、PPB1000具有介孔特性。当相对压力处于0.05~0.35范围内时,N_2_在生物炭质上发生可由BET方程描述的多分子层吸附;而当相对压力≥0.40时毛细孔凝聚,出现H4型迟滞环,这进一步表明所制备的生物炭质存在不规则的介孔结构。

**图1:**
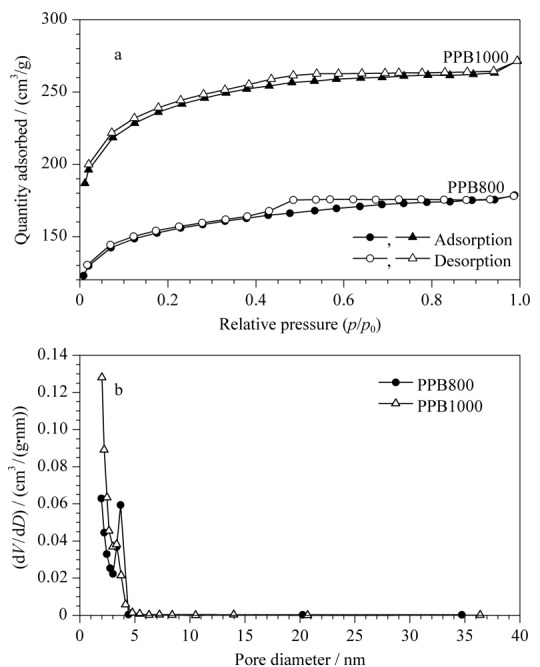
PPB800和PPB1000的(a) N_2_吸附-脱附等温线和(b)孔径分布图

从PPB800和PPB1000的N_2_吸附-脱附曲线可以看出:随着热解温度升高,PPB1000孔体积分布(0.42 cm^3^/g)和比表面积(749.9 m^2^/g)远高于PPB800的相关结构参数,这是因为柚子皮中的木质素、纤维素大部分被炭化,制得的PPB1000孔隙尺寸比PPB800更大。因此热解温度对生物炭质的结构参数起到关键作用。由孔径分布曲线(如图1b)可以看出,PPB1000和PPB800的孔径均在2~3 nm之间,远大于BTEX分子尺寸(直径约为0.60 nm),因此BTEX可以进入涂层的孔隙并与内部的官能团结合。

通过傅里叶变换红外光谱仪对PPB1000进行表征,其中处于4000~500 cm^-1^波数范围内的特征官能团的红外光谱图见图2a。由于果胶、纤维素和木质素是柚子皮的主要成分且这些成分主要包含醇酚醚等化合物,因此在3435 cm^-1^处的宽峰归属于-OH,该峰也可能源自N-H键的伸缩振动;1637 cm^-1^、611 cm^-1^两处存在的较强吸收峰,分别归属于C=O伸缩振动和-NH_2_的摇摆振动,表明该生物炭质中存在酰胺基团;1401 cm^-1^和813 cm^-1^处有峰存在,说明生物炭质中存在芳香环结构。FT-IR表征结果证明,在1000 ℃热解温度下得到的柚子皮生物炭质具有大量的官能团,这些官能团可以作为吸附结合位点通过*π-π*共轭和形成氢键有效去除水溶液中的BTEX。利用扫描电子显微镜(scanning electron microscope, SEM)对PPB1000的表面形貌进行了表征。从宏观图(如图2b)可以看到高温热解使得生物炭质表面呈现出粗糙的褶皱状表面结构,同时可以观察到其内部含有丰富的空腔。由柚子皮生物炭质的局部放大SEM图(如图2c)可以清楚看到生物炭质的孔道结构是由长而细的纤维状结构扭曲在一起形成的。这可能是由于热解温度较高,纤维素和木质素被分解,表面结构出现分解和坍塌所致。

**图2:**
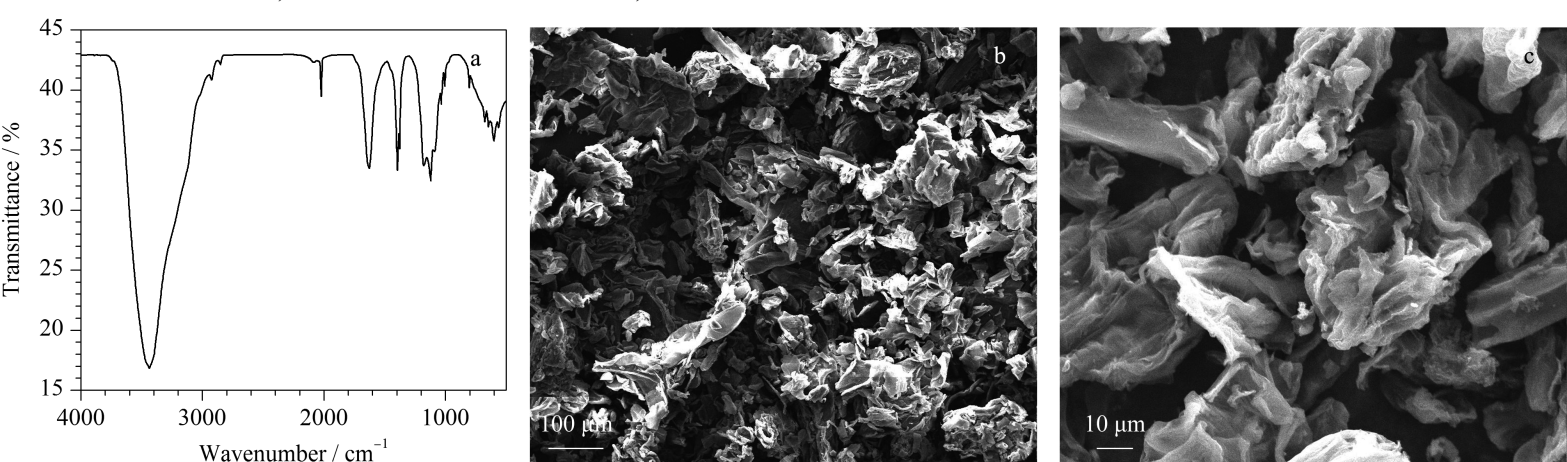
PPB1000的(a)红外光谱图和(b、c)扫描电镜图

### 2.2 萃取条件的优化

#### 2.2.1 解吸时间

萃取后,将纤维转移至GC进样口处。在GC进样器中,SPME纤维暴露于高温环境下,当温度到达分析物沸点时便发生解吸过程。在考察BTEX的沸点后(80~145 ℃),设定解吸温度为200 ℃。分别在0.5、1、2、3和4 min考察解吸时间对BTEX色谱峰面积的影响。如图3a所示,随着解吸时间增加至2 min,各BTEX的峰面积不断增大。而当解吸时间继续增加时,峰面积呈现减小的趋势,这可能是随着解吸时间增加,色谱峰发生变形所致。所以2 min被认为是BTEX在吸附剂中无残留的最佳解吸时间。

**图3:**
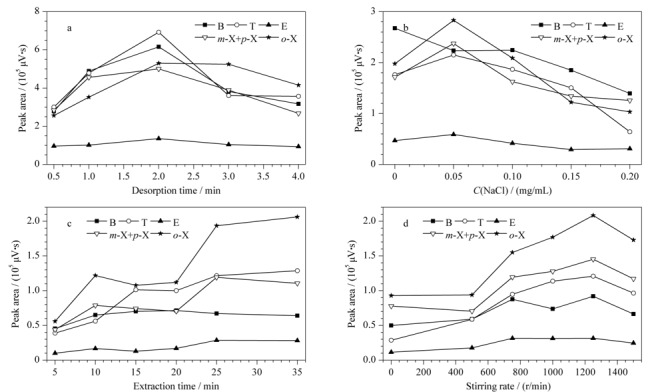
(a)解吸时间、(b)离子强度、(c)萃取时间和(d)搅拌速率对苯系物峰面积的影响

#### 2.2.2 离子强度

盐离子的添加会对萃取效率有双重影响。一般来说,盐离子的加入会降低目标分析物的溶解度,并增大它们在水溶液与涂层之间的分布系数。然而,对于浸入式固相微萃取(DI-SPME),过量盐离子的加入可能会降低分析物的萃取效率,因为盐会沉积在纤维涂层的表面并阻碍分析物的传质。实验考察了向15 mL样品溶液中加入不同质量的NaCl(溶液中NaCl的质量浓度为0~0.20 mg/mL)时的萃取效率。如图3b所示,从峰面积的走势曲线来看,盐离子的加入不利于苯的萃取,对于其他目标物,在当样品溶液中NaCl质量浓度为0.05 mg/mL时达到最大萃取量。因此实验最终选择向15 mL样品溶液中添加0.75 g NaCl,控制样品溶液中NaCl质量浓度为0.05 mg/mL。

#### 2.2.3 萃取时间

SPME是基于目标化合物在样品和萃取纤维之间的平衡过程。因此,需要确定最佳萃取时间。萃取过程在5~35 min之间进行,同时绘制目标分析物的峰面积与萃取时间关系图,如图3c所示:在15~35 min内,6种苯系物峰面积始终呈现增大的趋势。考虑到实际萃取效率,通常不需要达到传质平衡即可实现高灵敏分析。此外,当萃取时间超过20 min时,涂层会因吸附少量水溶液而造成FID熄火。因此最终萃取时间选为20 min。

#### 2.2.4 搅拌速率

对于SPME,应在纤维暴露期间对样品进行持续搅拌,以提高提取效率并最大限度地缩短提取时间。实验考察了在0~1500 r/min范围内的搅拌速度对BTEX峰面积的影响。如图3d所示,随着搅拌速度增加至1250 r/min, 6种BTEX的萃取量达到最大值,当搅拌速度超过1250 r/min时,样品瓶已不能稳定放置,故将搅拌速度设定为1250 r/min。

### 2.3 方法验证

在优化的萃取和色谱条件下,对1~100 μg/L范围内的BTEX混合标准溶液进行SPME实验,并用GC-FID进行分析来考察方法的线性范围、判定系数(*r*^2^)及检出限(LOD)等性能参数,相关结果列于表1。通过表1可以看出,在1~100 μg/L范围内,建立的方法对6种BTEX均呈现出良好的线性关系(*r*^2^≥0.9919)。各BTEX的LOD在0.004~0.032 μg/L之间,该方法可满足美国环境保护署规定的饮用水中BTEX最高限量的检测要求。为了进一步评估PPB1000的萃取能力,将其与商品化涂层聚二甲基硅氧烷(7 μm)进行了比较,由图4可以明显看出制备的材料对于BTEX的萃取能力明显高于商品化涂层,萃取效率约为商品化涂层聚二甲基硅氧烷(7 μm)的2.9~18.3倍,是SPME中一种有前途的吸附材料。

**表1:** 所建立方法的线性范围、检出限、判定系数以及精密度

Analyte	Linear range/ (μg/L)	LOD/ (μg/L)	r^2^	RSDs/%	
				Intra-batch (n=6)	Inter-batch (n=5)
B	1-100	0.032	0.9952	5.20	4.75
T	1-100	0.023	0.9948	1.04	1.03
E	1-100	0.020	0.9919	6.56	6.95
m-X+p-X	1-100	0.005	0.9975	5.76	12.42
o-X	1-100	0.004	0.9952	6.01	9.29

**图4:**
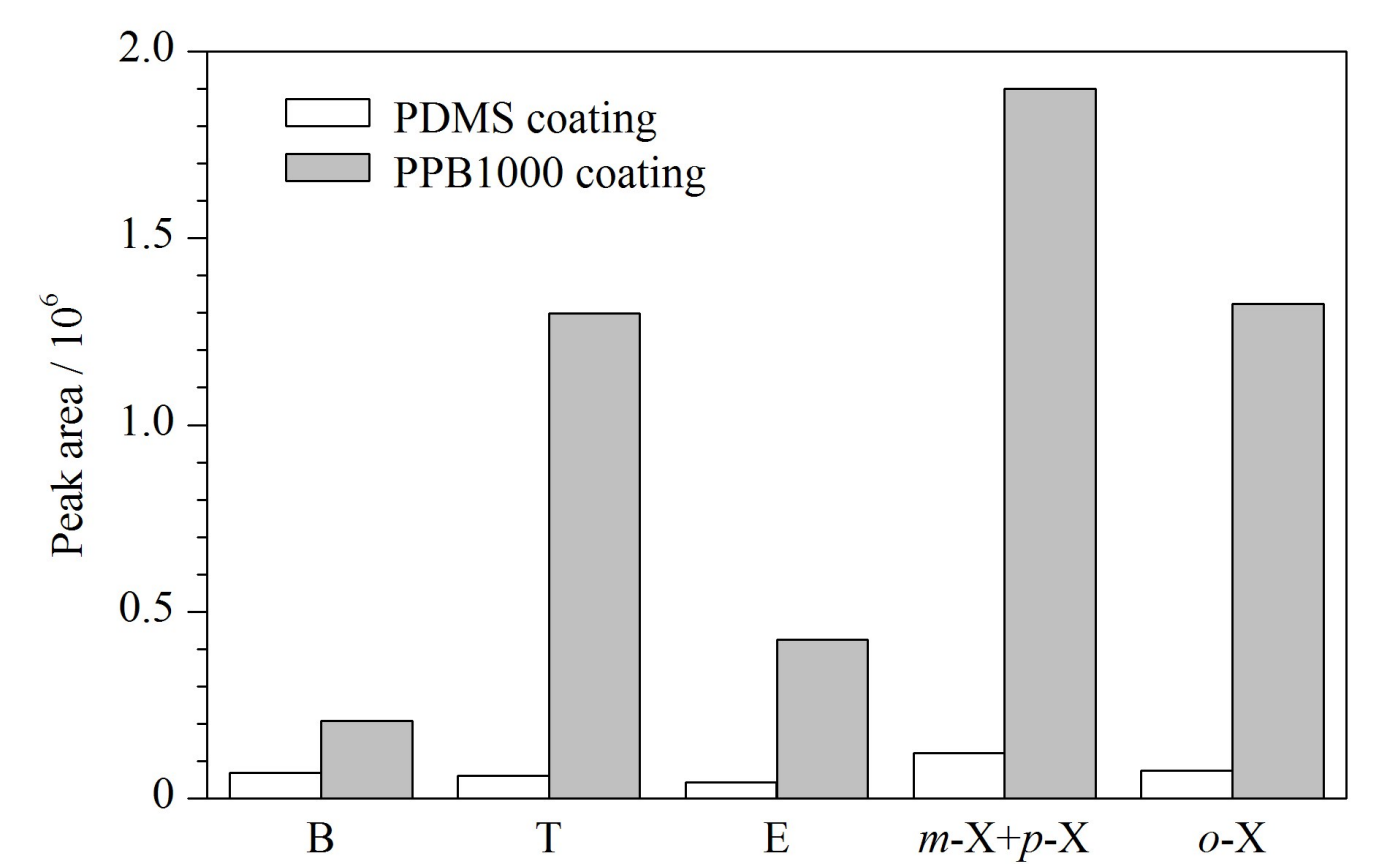
PPB1000涂层与聚二甲基硅氧烷涂层对苯系物萃取效率的比较

### 2.4 重复性和稳定性

就SPME技术在分析化学中的实际应用而言,萃取重复性和固定相稳定性是最重要的参数。使用同一根SPME纤维在最优实验条件下连续进行6次萃取后,涂层萃取效率没有明显下降,如表1所示,该方法具有良好的重复性(RSD≤6.56%)。此外,使用同一批次制备的柚子皮生物炭质粉末制备了5根SPME纤维进行实验,结果表明6种BTEX的RSD均≤12.42%,因此实验制备的SPME纤维可以保证实验结果的重复性。

该制备方法结合sol-gel良好的热化学稳定性以及不锈钢丝优异的机械强度,在多次萃取实验后,涂层没有发生明显的溶胀和脱落的现象,表明制备的PPB1000涂覆的SPME纤维具有良好的稳定性。

### 2.5 实际样品(河水)检测

通过分析实际水样(河水)中痕量BTEX,评估了所建立方法的适用性和实际应用性。在实际河水样品中成功检测出了乙基苯(4.80 μg/L),邻二甲苯(3.00 μg/L)和对二甲苯、间二甲苯(2.46 μg/L)(见图5)。实际水样中苯、甲苯的含量低于本方法的检出限。此外,考察了河水中不同加标水平下BTEX的回收率,以评价该分析方法的可靠性。由表2可以看出,加标水平为5、50、100 μg/L的河水样品,回收率在75.7%~117.6%范围内。以上实验结果表明所建立的分析方法具有良好的准确性,适用于环境水样中BTEX的日常监测。

**图5:**
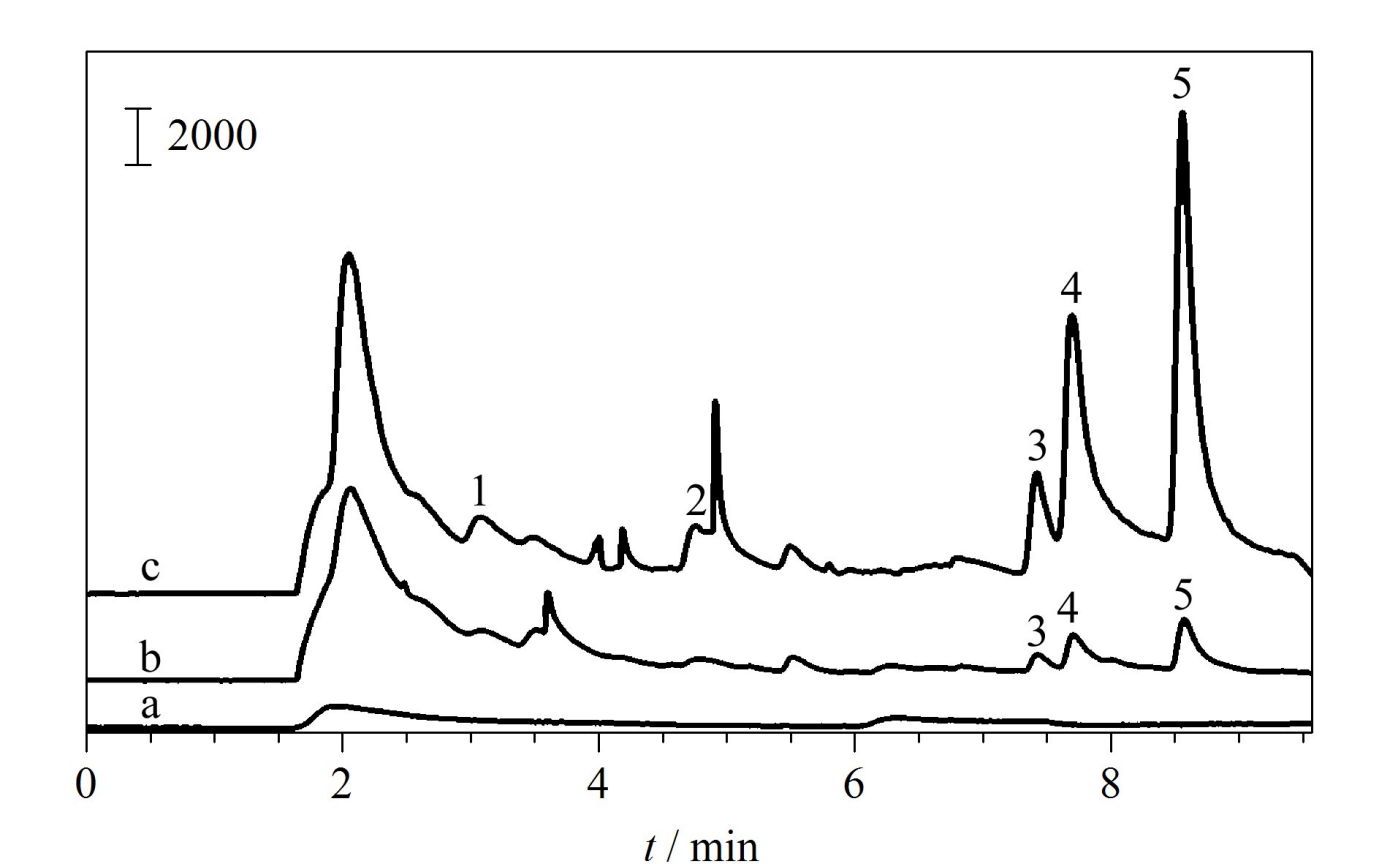
(a)PPB1000涂层、(b)空白河水样品和(c)加标河水样品(50 μg/L)的色谱图

**表2:** 6种苯系物在河水样品中的加标回收率

Analyte	Content/(μg/L)	Recoveries/%		
		5 μg/L	50 μg/L	100 μg/L
B	-	98.8	111.9	87.4
T	-	76.5	107.1	117.6
E	4.80	90.9	99.5	108.0
m-X+p-X	2.46	100.1	109.6	111.6
o-X	3.00	75.7	105.4	94.2

-: not detected.

## 3 结论

本文首先以柚子皮为原料制备了生物炭质PPB1000,将其作为绿色吸附剂,进一步通过sol-gel法,将其修饰在预处理过的不锈钢丝上制成SPME纤维。进一步与GC-FID相结合并对萃取和分离条件进行优化,建立了具有线性范围宽、灵敏度高的分析方法。该方法已在实际河水样品中成功检测出了乙基苯、对二甲苯、间二甲苯、邻二甲苯。综上,该方法不仅在涂层材料制备上绿色、简单、经济可行,同时适用于复杂水样基质中多种BTEX的分离检测。
